# Mechanistic Insights into Clinically Relevant Ribosome-Targeting Antibiotics

**DOI:** 10.3390/biom14101263

**Published:** 2024-10-07

**Authors:** Szymon J. Krawczyk, Marta Leśniczak-Staszak, Ewelina Gowin, Witold Szaflarski

**Affiliations:** 1 Department of Histology and Embryology, Poznan University of Medical Sciences, 60-781 Poznań, Poland; szymonkrawczyk627@gmail.com (S.J.K.); marta.m.lesniczak@gmail.com (M.L.-S.); 2 Department of Health Promotion, Poznan University of Medical Sciences, 60-781 Poznań, Poland; ewego@ump.edu.pl; 3 Department of Immunology, Poznan University of Medical Sciences, 60-806 Poznań, Poland

**Keywords:** protein biosynthesis, antibiotics, ribosome

## Abstract

Antibiotics targeting the bacterial ribosome are essential to combating bacterial infections. These antibiotics bind to various sites on the ribosome, inhibiting different stages of protein synthesis. This review provides a comprehensive overview of the mechanisms of action of clinically relevant antibiotics that target the bacterial ribosome, including macrolides, lincosamides, oxazolidinones, aminoglycosides, tetracyclines, and chloramphenicol. The structural and functional details of antibiotic interactions with ribosomal RNA, including specific binding sites, interactions with rRNA nucleotides, and their effects on translation processes, are discussed. Focus is placed on the diversity of these mechanisms and their clinical implications in treating bacterial infections, particularly in the context of emerging resistance. Understanding these mechanisms is crucial for developing novel therapeutic agents capable of overcoming bacterial resistance.

## 1. Introduction

The discovery of penicillin, the first widely used modern antibiotic, by Alexander Fleming revolutionized the treatment of bacterial infections [1]. Penicillin inhibits the enzyme responsible for cross-linking peptidoglycan in the bacterial cell wall, which is crucial for maintaining cell structure [2]. However, it quickly became apparent that other cellular components, such as ribosomes, responsible for protein biosynthesis, are also effective targets for antibiotics [3].

**Figure 1:**
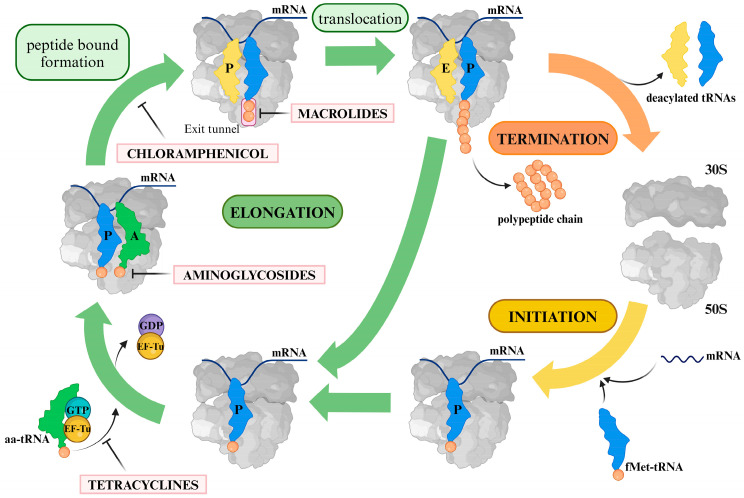
Antibiotics actions during protein synthesis.

Antibiotics targeting the bacterial ribosome fall into several classes, each with a specific mechanism of action. These antibiotics can be broadly categorized based on their binding sites on the ribosome and their effects on various stages of protein synthesis (Figure 1). The diversity of antibiotic mechanisms and their impact on bacterial ribosomes highlight the importance of understanding these drugs’ modes of action and clinical applications (Table 1).

### 1.1. Macrolides

Macrolides are a class of antibiotics characterized by a central lactone ring with attached side chains [4]. This ring structure typically contains 14, 15, or 16 carbon atoms. The composition and number of these side chains represent another distinguishing feature of the antibiotics in this group. The ring structure is critical for the antibiotic’s activity and interaction with the ribosomal RNA. The action of macrolides involves inhibiting translation by binding near the peptidyl transferase center of the large ribosomal subunit, which affects the ribosomal exit tunnel [5]. As a result, the emerging polypeptide cannot pass through the tunnel. Erythromycin, azithromycin, clarithromycin, and roxithromycin are clinically relevant macrolide antibiotics.

Erythromycin primarily targets the 50S ribosomal subunit, specifically binding to the peptidyl transferase center (PTC) within the 23S rRNA [6]. This binding occurs at the entrance of the nascent peptide exit tunnel, where erythromycin blocks the elongation of the growing polypeptide chain by physically obstructing its passage. The antibiotic interacts with critical nucleotides, such as A2058 and A2059, forming hydrogen bonds that stabilize its position [6]. The structural details revealed in these studies show that erythromycin binding does not cause significant conformational changes in the ribosome but inhibits translation by narrowing the tunnel, reducing its diameter to a size that prevents the exit of polypeptides longer than a few residues.

Initially, it was accepted that macrolides act as a physical barrier within the tunnel, effectively halting the elongation of all nascent proteins [7]. However, recent studies have demonstrated that macrolides do not uniformly inhibit the translation of all proteins but rather target a specific subset. It was discovered that particular amino acid sequences within the nascent peptide are more likely to cause ribosomal stalling when the ribosome is bound to an antibiotic [8]. These sequences are often referred to as arrest motifs. Upon recognition, the translation of the protein containing an arrest motif is inhibited explicitly in the presence of a drug. One example of such a motif is the sequence Lys/Arg-X-Lys/Arg, where X can be any amino acid, and this sequence is an arrest motif for erythromycin [8].

As a representative of macrolides, erythromycin exhibits a broad antibacterial spectrum [9], primarily targeting Gram-positive bacteria [10]. It is also effective against atypical bacteria (neither Gram-positive nor Gram-negative), including *Chlamydia* sp., *Mycoplasma* sp., and *Legionella* sp. Its activity against Gram-negative bacteria is more limited [11]. Erythromycin is rarely prescribed for patients—due to many adverse drug reactions. It carries the most prominent risk of cardiotoxicity among commonly used macrolide antibiotics [12]. Erythromycin is a motilin receptor agonist with more gastrointestinal side effects than other antibiotics [13]. It may also increase the risk of pyloric stenosis in the newborn [14].

**Table 1:** Clinically relevant antibiotics that target the prokaryotic ribosomes.

Antibiotic	The Primary Reference	Class	Ribosome Binding Site
Erythromycin	[6]	Macrolides	50S, PTC, exit tunnel
Azithromycin	[15]	Macrolides	50S, PTC, exit tunnel
Clarithromycin	[6]	Macrolides	50S, PTC, exit tunnel
Roxithromycin	[6]	Macrolides	50S, PTC, exit tunnel
Telithromycin	[16]	Ketolide	50S, PTC, exit tunnel
Amikacin	[17]	Aminoglycosides	30S, A-site
Gentamicin	[18]	Aminoglycosides	30S, A-site
Tobramycin	[19]	Aminoglycosides	30S, A-site
Netilmicin	[20]	Aminoglycosides	30S, A-site
Neomycin	[18]	Aminoglycosides	30S, A-site
Streptomycin	[21]	Aminoglycosides	30S, A-site, uS12
Linezolid	[22]	Oxazolidinones	50S, PTC
Clindamycin	[6]	Lincosamides	50S, PTC
Lincomycin	[23]	Lincosamides	50S, PTC
Chloramphenicol	[24]	Amphenicols	50S, PTC
Tetracycline	[25]	Tetracyclines	30S, A-site

Azithromycin, a 15-membered macrolide antibiotic, functions by binding to the large ribosomal subunit of bacteria, specifically within the peptide exit tunnel adjacent to the PTC [15]. Azithromycin’s binding site overlaps with other macrolides, such as erythromycin, but its structure allows for different interaction dynamics. The azithromycin molecule binds with its lactone ring oriented in a low-energy, folded-out conformation, with the hydrophobic face interacting with a hydrophobic patch on the tunnel wall. The desosamine sugar moiety of azithromycin extends toward the PTC, contributing to its inhibitory action by physically obstructing the exit tunnel. This blockage prevents the elongating polypeptide from passing through, effectively halting protein synthesis after adding a few amino acids. Additionally, the binding of azithromycin induces conformational changes in specific rRNA nucleotides, such as A2058 and A2059 in *E. coli*, further stabilizing its binding and enhancing its inhibitory effect [26].

Azithromycin exhibits a broad antibacterial spectrum, like erythromycin, with enhanced activity against Gram-negative bacteria [27]. Its extended half-life permits once-daily dosing and a shorter course of treatment than other macrolides (from 1 to 5 days). Azithromycin is a safe antimicrobial agent with only a few adverse effects. It is also considered safer and has fewer cardiac adverse effects than other macrolides [27].

**Figure 2:**
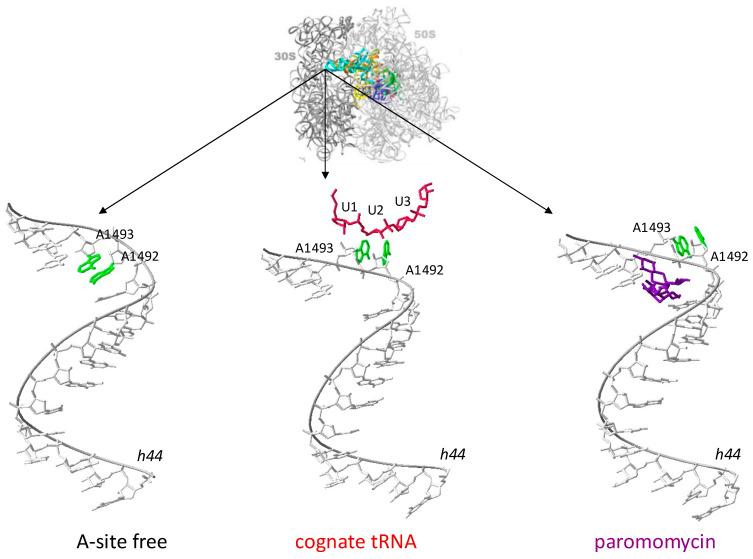
The location of adenine residues when the A-site is unoccupied by any aa-tRNA (A-site free); when cognate aa-tRNA is bound to the A-site (cognate tRNA), which causes the flipping out of residues A1492 and A1493; and finally, when binding of paromomycin artificially flips out residues A1492 and A1493. PDB accession numbers are as follows: 1J5E (A-site free) [21], 1IBM (cognate tRNA) [28], and 1IBL (paromomycin) [28]. Green—adenine residues, red—anticodon stem-loop of the cognate tRNA, purple—paromomycin molecule.

Like other macrolides, clarithromycin binds to the bacterial ribosome’s large subunit, specifically within the peptide exit tunnel near the PTC of the 23S rRNA [6]. Clarithromycin binds in the tunnel in what is referred to as a “folded-in” conformation, which distinguishes it from larger-ring macrolides, like azithromycin, that bind in a “folded-out” conformation. In this orientation, the lactone ring of clarithromycin positions itself almost perpendicularly to the tunnel wall, and the cladinose sugar moiety forms unique interactions that stabilize the drug within the ribosome. This configuration allows for clarithromycin to effectively block protein synthesis by halting the elongation of the polypeptide chain, thereby inhibiting bacterial growth and division [29].

Clarithromycin acts against a broad spectrum of Gram-positive, Gram-negative, and atypical bacteria. The profile of side effects is like that of other macrolides. Highly stable in the presence of gastric acid (unlike erythromycin), it results in better absorption after oral intake [30,31].

The roxithromycin binding site is located at the peptide exit tunnel’s entrance, a channel through which newly synthesized proteins exit the ribosome. The fundamental interactions that stabilize roxithromycin in its binding site involve hydrogen bonds between the hydroxyl groups on the lactone ring and the desosamine and cladinose sugars in the antibiotic’s structure with specific nucleotides in the 23S rRNA, such as A2058, A2059, and G2505 [6]. These interactions are crucial for maintaining the antibiotic’s position and effectiveness in inhibiting the exit tunnel. Furthermore, like other macrolides, roxithromycin may also influence the rRNA’s conformational stability, further enhancing its antibacterial activity.

Roxithromycin exhibits activity against a broad spectrum of Gram-positive, Gram-negative, and atypical bacteria [32,33]. It has fewer drug interactions than erythromycin.

Telithromycin is not a typical macrolide because it features a modified chemical structure, specifically a ketone group, which replaces the cladinose sugar found in traditional macrolides, making it a ketolide [34]. This modification allows for telithromycin to bind to additional sites on the ribosome, enhancing its effectiveness against bacteria resistant to classic macrolides and reducing its susceptibility to bacterial resistance mechanisms. The primary binding site is located at the PTC, where, like other macrolides, telithromycin binds at the entrance of the peptide exit tunnel within the 23S rRNA [16]. This interaction involves hydrogen bonding between the drug and specific nucleotides within the rRNA, notably A2058 and A2059. These adenine nucleotides are crucial for binding macrolides, with the N6 atom of A2058 playing a significant role by typically forming a hydrogen bond with the 2′-OH group of the desosamine sugar in macrolides. Telithromycin’s interaction with these nucleotides is crucial in blocking the exit tunnel and preventing the elongation of the nascent polypeptide chain [5].

Additionally, the guanine nucleotide G2505 serves as another critical contact point, helping to stabilize telithromycin’s binding within the ribosomal tunnel [35]. Beyond this traditional macrolide binding site, telithromycin also binds at a secondary site within the ribosome, unique to ketolides. This secondary site involves interactions with nucleotides in domain II of the 23S rRNA. The dual binding mechanism enhances telithromycin’s inhibitory effect. It reduces the likelihood of bacterial resistance, as mutations in the ribosomal RNA that confer resistance to macrolides may not significantly affect this secondary binding site.

Telithromycin, the first ketolide antibiotic, exhibits a broad spectrum of activity, particularly against respiratory pathogens. It is highly effective against Gram-positive bacteria, including *Streptococcus pneumoniae*, and even strains resistant to macrolides (due to erm (B) or mef (A) genes) and penicillin [34]. Additionally, telithromycin is potent against atypical respiratory pathogens and some Gram-negative respiratory bacteria [36].

### 1.2. Lincosamides

Lincosamides are another class of antibiotics with a mechanism of action like macrolides. However, lincosamides do not contain a lactone ring but instead consist of a 1-propyl hydronic acid and an eight-carbon amino sugar, which are linked together by an amide bond. Lincosamides act as structural analogues of a peptidyl tRNA and a deacylated tRNA at an initial stage of pre-translocation [37]. Two different lincosamides are used in medical practice: lincomycin and its derivative, clindamycin.

Lincomycin and its semisynthetic derivatives act by specifically binding to the large ribosomal subunit (50S) of bacteria [38]. More precisely, they bind close to the PTC, which blocks the PTC itself. Furthermore, this binding blocks peptide bond formation, thereby preventing the proper positioning of the A-site tRNA and inhibiting protein synthesis. Structural studies have shown that the propylhygric acid moiety of lincomycin occupies the PTC, interfering with the accommodation of the A-site’s 3′ ends of tRNA. In contrast, the sugar moiety (β-MTL) forms a critical network of hydrogen bonds with 23S rRNA nucleotides, such as A2058, A2059, and G2505, stabilizing the ribosomal complex [23]. A novel semisynthetic derivative, RB02, differs from lincomycin by the presence of a para-nitrobenzamide group, which reduces steric hindrance but also weakens its interactions with the ribosome, resulting in lower efficacy in inhibiting translation, particularly in *E. coli* ribosomes [23]. Additionally, spermidine, observed in the lincomycin binding site, may stabilize these interactions by binding to nucleotide A2062, suggesting that spermidine plays a role in stabilizing the antibiotic–ribosome complex. It has been shown that nucleotide A2062 can adopt different conformations depending on the presence of various lincosamides, which may significantly impact their ability to inhibit the ribosome. Despite the overall structural similarities, differences in the conformation and specificity of binding among different lincosamide derivatives highlight the complexity of their structural mechanism of action.

Lincomycin is active against Gram-positive bacteria and against anaerobic bacteria. However, it is essential to note the cross-resistance between lincomycin and macrolides (MLs), which may limit its efficacy in treating certain bacterial infections, particularly in pathogens that exhibit resistance to this class of antibiotics [39].

Clindamycin is a semisynthetic derivative of lincomycin, distinguished by the presence of a chlorine atom at the C7 position and inverted stereochemistry at that site. Like other lincosamides, clindamycin binds to the large ribosomal subunit (50S) of bacteria, blocking the PTC and preventing the proper positioning of tRNA at the A-site [6]. A previous study highlights that clindamycin exhibits higher clinical potency than lincomycin, particularly against Gram-positive bacteria [40]. This may be attributed to improved pharmacokinetic properties, such as enhanced cell membrane permeability, rather than significant differences in ribosomal binding interactions.

Structurally, clindamycin’s binding interactions with the ribosome are like those of lincomycin, involving forming hydrogen bonds with critical 23S rRNA nucleotides within the PTC. However, due to the presence of the chlorine atom and the altered C7 stereochemistry, clindamycin may influence the stability of the ribosomal complex differently. Notably, clindamycin lacks specific interactions with nucleotide A2062 that are observed with lincomycin, potentially due to the steric effects of the chlorine substitution. While not significantly altering the drug’s inhibitory effect on the ribosome in vitro, this structural variation suggests that clindamycin’s enhanced clinical efficacy may result from factors beyond its direct interaction with the ribosome, underscoring the complexity of its action mechanism [41].

Clindamycin exhibits broad antimicrobial activity, particularly against Gram-positive bacteria such as *Streptococcus pyogenes*, *Streptococcus pneumoniae* (with PR strains potentially susceptible under the M phenotype), and *Staphylococcus aureus*, including MRSA strains, although cross-resistance with macrolides may occur [42]. It is also effective against anaerobes. Beyond its antibacterial properties, clindamycin demonstrates activity against certain protozoa—*Toxoplasma gondii* [43].

### 1.3. Oxazolidinones

Oxazolidinones are antibiotics characterized by the presence of a 1,3-oxazolidin-2-one skeleton [44]. They bind to the A-site of the large ribosomal subunit, the same binding site as lincosamides, which means these compounds compete for ribosome binding. By binding to this site, oxazolidinones inhibit translation in two ways: they either hinder the translation initiation complex from associating with mRNA [22], or, if the initiation complex successfully attaches, they block elongation by preventing the tRNA from transitioning from the A-site to the P-site within the ribosome [22].

Linezolid, a pioneering oxazolidinone antibiotic, exerts its antibacterial activity by selectively binding to the peptidyl transferase center (PTC) of the 50S ribosomal subunit, a critical site for protein synthesis. Structural analysis reveals that linezolid’s oxazolidinone ring interacts with the base of U2539. At the same time, its C-5 acetamide arm forms a hydrogen bond with the phosphate group of G2540, effectively anchoring the molecule within an all-RNA pocket of the PTC. The fluorophenyl moiety of linezolid further stabilizes this binding through aromatic interactions with A2486 and C2487, positioning the antibiotic in a way that competes directly with A-site substrates. This binding disrupts the correct positioning of aminoacyl-tRNA [22]. It induces significant conformational changes in the PTC, particularly the rotation of U2539 and C2487, which optimize the drug’s fit and enhance its inhibitory effect. Moreover, a study elucidates the molecular basis of resistance, where mutations such as G2611U disrupt the structural integrity of the binding pocket, diminishing linezolid’s affinity and leading to resistance. These insights into linezolid’s mechanism of action and the structural adaptations of the ribosome underscore the potential for designing next-generation oxazolidinones that can overcome emerging resistance, thereby extending the clinical utility of this vital antibiotic class [45,46].

Linezolid is effective against Gram-positive and some anaerobic bacteria [47]. It is not effective against Gram-negative bacteria. Linezolid is toxic to mitochondria—data suggest that linezolid interferes with mitochondrial protein synthesis due to similarities between bacterial and mitochondrial ribosomes [48]. This results in lactic acidosis and peripheral neuropathy [49].

### 1.4. Aminoglycosides

Aminoglycosides are a powerful class of antibiotics that disrupt bacterial protein synthesis by targeting the 30S ribosomal subunit with wide medical applications [50]. The core mechanism involves binding to specific sites on the ribosomal RNA (rRNA) within the 16S rRNA component, leading to errors in the translation process [51]. While all aminoglycosides share this general mode of action, each antibiotic’s specific interactions and structural nuances result in varied effects on the ribosome and, consequently, on the bacterial cell.

Paromomycin (without applying in the medicine), gentamicin, tobramycin, and netilmicin are prime aminoglycosides that bind predominantly to helix 44 (h44) in the 16S rRNA [28]. These antibiotics have a binding site within two adenine moieties (A1492 and A1493) within h44 (Figure 2). The binding of paromomycin artificially flips out these two residues, which promotes aa-tRNA binding to the A-site without correct ribosomal proofreading. It causes the insertion of incorrect aa-tRNA and the incorporation of amino acid into the nascent polypeptide chain, which is not codded by a codon on mRNA. This misreading causes the synthesis of aberrant proteins that are often nonfunctional or harmful to the bacterial cell, contributing to cellular dysfunction and, ultimately, cell death [52,53].

In the case of gentamicin, its primary interaction site within h44 causes translational errors by stabilizing the mispairing of codon–anticodon interactions. However, gentamicin also can bind to a secondary site on the 50S ribosomal subunit near the H69 bridge [28,54]. This additional binding site affects the ribosome’s ability to recycle after a round of translation, further impairing protein synthesis by disrupting the termination and recycling stages. The interaction at this secondary site may involve conformational changes in the rRNA that promote the binding of near-cognate tRNA, thereby exacerbating the frequency of translational errors.

Tobramycin, structurally like gentamicin, also binds to h44 and induces similar disruptions in the decoding process. However, subtle differences in the chemical structure of tobramycin, such as variations in the hydroxyl and amino groups attached to the aminocyclitol ring, may influence its binding affinity and the precise nature of its interactions with the ribosome [19]. These structural differences can affect how efficiently tobramycin induces errors at the decoding site and may contribute to its distinct antibacterial spectrum compared to gentamicin and amikacin [17].

Netilmicin, another aminoglycoside closely related to gentamicin, is chemically modified with a methyl group at the N1 position, enhancing its resistance to bacterial enzymes that typically inactivate other aminoglycosides [20]. This structural modification broadens netilmicin’s spectrum of activity, particularly against resistant bacterial strains, and slightly alters its binding dynamics within the ribosome. While netilmicin primarily targets h44, like gentamicin and tobramycin, the presence of the methyl group may influence its interaction with adjacent rRNA regions, potentially offering a slightly different inhibition profile.

In contrast to these aminoglycosides, streptomycin exhibits a distinct mechanism of action despite also targeting the 30S ribosomal subunit. Streptomycin binds not only to helix 44 but also forms significant interactions with the G530 loop of the 16S rRNA and the ribosomal protein uS1 [21]. The G530 loop and uS12 are crucial elements in the decoding site of the ribosome, and streptomycin’s interaction with these components induces a unique conformational change that alters the geometry of the A-site. This conformational change significantly disrupts the accuracy of tRNA selection, causing a more profound increase in translational errors compared to neomycin or other aminoglycosides that primarily affect h44 [21]. By distorting the decoding center’s structure, streptomycin forces the ribosome to accept non-cognate tRNAs at a higher rate, leading to the incorporation of incorrect amino acids more frequently than with other aminoglycosides.

Neomycin, another aminoglycoside, binds similarly to gentamicin and tobramycin, targeting h44 and causing misreading of the genetic code [55]. However, it does not interact with the G530 loop or uS12 as streptomycin does, which results in a slightly less disruptive impact on the ribosome’s fidelity. Neomycin’s primary mechanism of action involves inducing translational errors through its interaction with h44, producing dysfunctional proteins. These proteins can accumulate in the bacterial cell, causing stress and ultimately leading to cell death.

The structural differences between these aminoglycosides determine their specific mechanisms of action and influence their antibacterial spectrum and the development of bacterial resistance. For instance, the modifications seen in netilmicin provide a protective advantage against enzymatic degradation. At the same time, the unique interactions of streptomycin with the G530 loop and uS12 confer a distinct pattern of ribosomal inhibition. These variations highlight the adaptability and complexity of aminoglycosides as a class, with each drug leveraging specific structural features to achieve its antibacterial effects while facing unique challenges related to bacterial resistance mechanisms.

Aminoglycosides exhibit diverse activity against Gram-positive and Gram-negative bacteria [56] but no activity against anaerobic cocci [57]. Their efficacy against Gram-positive bacteria, particularly streptococci and staphylococci, can be enhanced when combined with β-lactam antibiotics [51,58].

### 1.5. Tetracyclines

**Figure 3:**
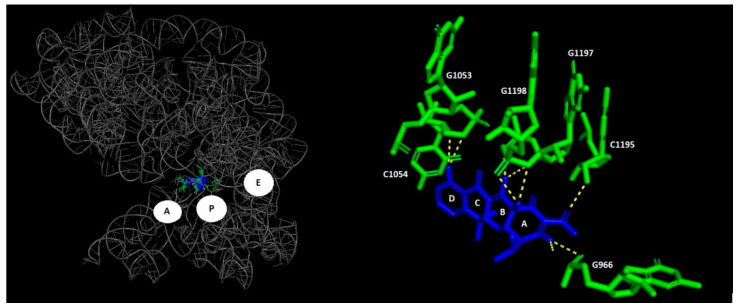
The binding site of tetracycline. Left panel: A, P, and E functional sites (grey—rRNA, blue—tetracycline, green—tetracycline-interacting rRNA). Right panel: tetracycline shown in blue; surrounding rRNA demonstrated in green (PDB accession no. 1I94).

Tetracyclines are a class of antibiotics characterized by a conserved core structure of four fused hydrocarbon rings [59]. These antibiotics function primarily by inhibiting bacterial protein synthesis through their interaction with the 30S ribosomal subunit. Tetracyclines bind to the A site of the 16S rRNA within the 30S subunit, explicitly interacting with nucleotides in helix 34 (Figure 3). This binding blocks the aminoacyl-tRNA (aa-tRNA) attachment to the mRNA–ribosome complex, effectively preventing adding new amino acids to the elongating polypeptide chain [25]. The interruption of this crucial step in protein synthesis leads to the cessation of bacterial growth and, ultimately, cell death.

The detailed structural analysis using cryo-electron microscopy reveals that tetracyclines interact with the ribosome through multiple direct and indirect hydrogen bonds. Direct interactions include at least six hydrogen bonds formed with the backbone of rRNA nucleotides in helix 34 [60]. A key feature of tetracycline binding is the involvement of magnesium ions, which are essential for stabilizing the antibiotic–ribosome interaction. One primary magnesium ion is coordinated by oxygen atoms from the tetracycline molecule and surrounding rRNA nucleotides, forming a stable octahedral complex. Additionally, oxygen atoms from water molecules and rRNA coordinate a secondary magnesium ion, further enhancing the stability of the tetracycline binding.

Interestingly, water molecules play a significant role in mediating the interactions between tetracyclines and the ribosome. These water molecules are crucial for coordinating magnesium ions and participate in a network of hydrogen bonds that stabilize the antibiotic within its binding site. The study shows that upon tetracycline binding, two water molecules coordinating Mg are displaced, allowing the drug to fully engage with the ribosomal RNA, thereby enhancing its inhibitory effect.

Compared to other tetracycline derivatives, such as omadacycline and eravacycline, the common structural core in all these molecules exhibits a high degree of structural conservation in its binding mode to the ribosome [61]. However, subtle differences in the conformation around the A ring of the tetracycline scaffold influence how the magnesium ion (Mg^2+^) is coordinated. For example, in omadacycline and pentacycline, the conformation allows for direct coordination of Mg^2+^ by phosphate oxygens in the rRNA. In contrast, in tetracycline and eravacycline, this coordination occurs indirectly via water molecules. Despite these differences, the overall mechanism of action remains conserved across the tetracycline class, emphasizing the critical role of magnesium and water-mediated interactions in their function.

The high-resolution structural data suggest that tetracyclines’ extensive hydrogen bonding and coordination networks contribute significantly to their binding affinity and specificity. This detailed understanding of tetracycline–ribosome interaction provides insights into the mechanism of action of these antibiotics. It offers valuable information for the design of next-generation tetracyclines that can overcome emerging bacterial resistance [61].

Tetracyclines exhibit a broad spectrum of antibacterial activity, targeting all types of bacteria (Gram-positive, Gram-negative, and atypical) [62]. Notably, some MRSA strains remain sensitive to doxycycline, broadening tetracyclines’ clinical utility against these resistant organisms. Tetracyclines are also active against Gram-negative bacteria, though high resistance levels are also observed [63]. Resistance mechanisms primarily involve efflux pumps and ribosomal protection proteins. However, enzymatic inactivation of tetracyclines, particularly among anaerobic bacteria like *Bacteroides* spp., also contributes to resistance.

### 1.6. Chloramphenicol

Chloramphenicol targets the ribosome’s large subunit, specifically the PTC, in bacterial cells. Its mechanism of action is based on binding to the 23S rRNA of the 50S ribosomal subunit, inhibiting peptide bond formation, which prevents the growth of the nascent polypeptide chain and halts protein synthesis [24]. Structurally, chloramphenicol binds in the A-site cleft of the PTC, where it directly competes with the incoming aminoacyl-tRNA, obstructing its proper accommodation and preventing peptide bond formation.

One key aspect of chloramphenicol’s action is that it exhibits context specificity. It preferentially stalls ribosomes when sequences of nascent peptides are present [64]. This is due to interactions between the drug and the penultimate residues of the nascent peptide. For example, when alanine, serine, or threonine residues are in the penultimate position, chloramphenicol is more likely to cause ribosome stalling. These residues establish specific interactions with chloramphenicol, such as hydrogen bonding or CH–π interactions, which stabilize the drug’s binding in the PTC. In contrast, bulkier residues like phenylalanine do not support chloramphenicol binding due to steric clashes, reducing inhibitory effects.

Thus, the drug’s binding pocket is not static but is influenced by the growing polypeptide chain, making chloramphenicol’s inhibitory action dependent on the specific sequence of the synthesized peptide [65].

Chloramphenicol exhibits a broad antimicrobial spectrum, effective against nearly all bacteria except *Pseudomonas aeruginosa*, *Acinetobacter* species, and *Nocardia [66]*.

## 2. General Remarks on the Resistance Mechanisms Associated with Clinically Relevant Antibiotics

Resistance mechanisms for antibiotics can vary depending on the class and specific drug. For macrolides like erythromycin, azithromycin, clarithromycin, roxithromycin, and telithromycin, the most common resistance mechanisms include modification of the target site via methylation of 23S rRNA, often encoded by the *erm* gene, and efflux pumps that expel the drug from the bacterial cell, mediated by *mef(A)* genes [39]. Telithromycin, a ketolide, is typically more effective than other macrolides due to a stronger affinity for ribosomes; however, resistance can still occur through mutations in domain V of the 23S rRNA [67]. Aminoglycosides such as amikacin, gentamicin, tobramycin, netilmicin, neomycin, and streptomycin encounter resistance primarily via enzymatic modification of the drug by aminoglycoside-modifying enzymes (AMEs), which phosphorylate, acetylate, or adenylate the antibiotic [68]. Additionally, changes in the bacterial ribosomal binding site and decreased uptake through membrane alterations can contribute to resistance. Linezolid, a member of the oxazolidinone class, faces resistance due to mutations in the 23S rRNA and acquisition of the *cfr* gene, which methylates the ribosomal binding site, preventing drug action [69]. Clindamycin and lincomycin, which belong to the lincosamide class, encounter resistance via methylation of the 23S rRNA, often through the *erm* gene, similar to macrolides, and enzymatic inactivation by lincosamide nucleotidyltransferases [70]. Chloramphenicol, while less frequently used today, is often resisted by producing chloramphenicol acetyltransferase (CAT), which inactivates the drug [71]. Finally, tetracycline resistance is most mediated by efflux pumps (e.g., tet(A) and tet(K)) and ribosomal protection proteins like Tet(M), which prevent tetracycline from binding to the ribosome, allowing for protein synthesis to continue [72].

## 3. Conclusions

The evolution of bacteria towards antibiotic resistance necessitates the search for new compounds that can effectively kill or inhibit bacterial growth without damaging host cells. One of the most promising areas of research is the discovery of new inhibitors of the bacterial ribosome. The ribosome, the essential machinery responsible for protein synthesis, presents an attractive target for antibiotics. Translation inhibitors that bind to the ribosome can stop bacterial growth by preventing the production of new proteins.

The development of new antibiotics often focuses on identifying compounds that selectively bind to bacterial ribosomes, inhibiting their function without affecting the host’s ribosomes. The discovery of compounds like chloramphenicol, which blocks the peptidyl transferase center in bacterial ribosomes, offers valuable insights for designing future drugs. The primary goal is to continue searching for inhibitors that act selectively and precisely, particularly against antibiotic-resistant bacteria.

Understanding the structural and molecular mechanisms by which these inhibitors function and their interactions with various peptide sequences synthesized by bacteria could lead to the development of new generations of antibiotics. This field has the potential to bring breakthroughs in the fight against drug resistance and in developing more effective antibacterial therapies.
